# Evolutionary hallmarks of the human proteome: chasing the age and coregulation of protein-coding genes

**DOI:** 10.1186/s12864-016-3062-y

**Published:** 2016-10-25

**Authors:** Katia de Paiva Lopes, Francisco José Campos-Laborie, Ricardo Assunção Vialle, José Miguel Ortega, Javier De Las Rivas

**Affiliations:** 1 Bioinformatics and Functional Genomics Group, Cancer Research Center (CiC-IBMCC, CSIC/USAL/IBSAL), Consejo Superior de Investigaciones Cientificas (CSIC), Salamanca, Spain; 2 Departamento de Bioquímica e Imunologia, Instituto de Ciências Biológicas (ICB), Universidade Federal de Minas Gerais (UFMG), Belo Horizonte, Brasil

**Keywords:** Human protein evolution, Human gene evolution, Transcriptomics, RNA-seq, Tissue transcriptomics, Protein families, Gene coexpression, Gene house-keeping, Gene tissue-enriched

## Abstract

**Background:**

The development of large-scale technologies for quantitative transcriptomics has enabled comprehensive analysis of the gene expression profiles in complete genomes. RNA-Seq allows the measurement of gene expression levels in a manner far more precise and global than previous methods. Studies using this technology are altering our view about the extent and complexity of the eukaryotic transcriptomes. In this respect, multiple efforts have been done to determine and analyse the gene expression patterns of human cell types in different conditions, either in normal or pathological states. However, until recently, little has been reported about the evolutionary marks present in human protein-coding genes, particularly from the combined perspective of gene expression and protein evolution.

**Results:**

We present a combined analysis of human protein-coding gene expression profiling and time-scale ancestry mapping, that places the genes in taxonomy clades and reveals eight evolutionary major steps (“hallmarks”), that include clusters of functionally coherent proteins. The human expressed genes are analysed using a RNA-Seq dataset of 116 samples from 32 tissues. The evolutionary analysis of the human proteins is performed combining the information from: (i) a database of orthologous proteins (OMA), (ii) the taxonomy mapping of genes to lineage clades (from NCBI Taxonomy) and (iii) the evolution time-scale mapping provided by TimeTree (Timescale of Life). The human protein-coding genes are also placed in a relational context based in the construction of a robust gene coexpression network, that reveals tighter links between age-related protein-coding genes and finds functionally coherent gene modules.

**Conclusions:**

Understanding the relational landscape of the human protein-coding genes is essential for interpreting the functional elements and modules of our active genome. Moreover, decoding the evolutionary history of the human genes can provide very valuable information to reveal or uncover their origin and function.

**Electronic supplementary material:**

The online version of this article (doi:10.1186/s12864-016-3062-y) contains supplementary material, which is available to authorized users.

## Background

RNA-Seq allows the measurement of the gene expression levels in a manner far more precise than previous methods. Studies using this approach have already altered our view of the extent and complexity of the eukaryotic transcriptomes [1]. Important analyses have been performed based on transcriptomic data obtained from multiple human tissues. The FANTOM project, for example, presents the gene expression profiling of 56 human healthy tissues associated with the functional annotation of the mammalian genomes [2]. The Human Protein Atlas applies RNA-Seq to 32 human tissues to find the correlation between gene expression and protein presence in the characterization of several parts of the human proteome: membrane proteome, druggable proteome, cancer proteome, secretome and proteome involved in metabolic processes [3]. The Genotype-Tissue Expression (The GTEx Consortium) [4] provides an extensive resource of human transcriptomic data permitting the inference of different patterns across human tissues and individuals [5], and the construction of tissue-specific gene co-expression networks [6]. Other studies are more focused on specific cells or tissue types, like the article entitled “*A comprehensive analysis of the human placenta transcriptome*” that characterizes the transcriptome of placenta from 20 healthy women with uncomplicated pregnancies using RNA-Seq [7].

The assembly of comprehensive maps of the human transcriptome is essential for a clear identification of the functional elements of our genome and to reveal the molecular constituents of different cells and tissues [1]. Despite the accomplishment of many transcriptomic studies, little has been reported until recently about the evolutionary determinants of human cell identity, particularly from a joint perspective of protein evolution and gene expression [8]. The evolutionary history of a gene can be very informative about its function. Gene age (i.e. the question *How old is a gene?*) is an important piece of information that can be inferred in different ways and has been used in some genome-scale studies and in some studies on gene families [9]. *Phylostratigraphy* is the usual methodology applied to find the origin and emergence of genes [10, 11]. Previous phylogenetic studies showed that the evolutionary history of different coding parts of the genome have relations with diseases [12], codon usage [13], essentiality, interactions [14], stemness and self-renewal [15]. Other studies have shown that older genes evolve slower [16], encode longer proteins, present higher expression levels, possess higher intron density and are subject to stronger purifying selection [17, 18]. Several of these studies approach the question of *gene age* in different ways, but most of them are not focused on human genes or do not applied phylostratigraphy using large-scale genomic data.

In this work, we address the key question about the age of the human genes using a combination of complementary genome-wide data and databases that are analysed and integrated to achieve a map of the human genes on the evolutionary time-scale. This integrative analysis uses a comprehensive human tissue RNA-Seq dataset that allows deep expression profiling of protein-coding genes [3]; a database of orthologous proteins that allows finding the oldest relatives to each human protein along different species [19, 20]; the taxonomy mapping of genes to lineage clades from the NCBI Taxonomy database (www.ncbi.nlm.nih.gov/taxonomy); and the time-scale mapping provided by TimeTree resource (www.timetree.org) [21, 22]. The generation of clusters of orthologous proteins built along multiple species is more accurate for phylogenetic studies than simple sequence homology search [19], because it does not look for singular best homologous but it implies a conservation along the evolutionary tree and can provide a way to date the origin of protein modules involved in specific functions or in specific biological pathways [23, 24]. Finally, to complete the view of the protein-coding gene phylostratigraphy, we use the genome-wide expression data to produce a human gene network based on a coexpression analysis of the transcriptomic RNA-Seq profiles along multiple tissues and identify the genes that can be considered *House-keeping* (HKg) or *Tissue-enriched* (TEg). The allocation of these gene subsets (HKg and TEg) on the evolutionary time map shows a clear difference in gene age, indicating that house-keeping genes are older.

## Methods

### Gene expression data from human normal tissues

The genome-wide expression dataset used in this work corresponds to a series of RNA-Seq analyses performed with Illumina HiSeq 2000 paired end sequencing on cDNA libraries prepared from samples of 122 human individuals from 33 different tissues [3] (ArrayExpress DB: E-MTAB-2836). The data provided reads for 20,344 genes detected per sample, and 18,545 of these genes showed relevant expression signal corresponding to mean(FPKM) ≥ 1 in all the selected tissues. After normalization and comparative analysis of the expression distributions of the samples from this dataset, we selected a total of 116 samples with two to five biological replicates for the following 32 tissues: adrenal gland (3 replicates), appendices (3), bone marrow (4), brain (3), colon rectum (5), duodenum (2), endometrium (5), esophagus (3), fallopian tube (5), adipose tissue (3), gallbladder (3), heart (4), kidney (4), liver (3), lung (5), lymph node (5), ovary (3), pancreas (2), placenta (4), prostate (4), rectum (4), salivary gland (3), skeletal muscle (5), skin (3), small intestine (4), smooth muscle (3), spleen (4), stomach (3), testis (5), thyroid (4), tonsil (3) and urinary bladder (2).

### Expression profiling and coexpression data analyses

**Fig. 1:**
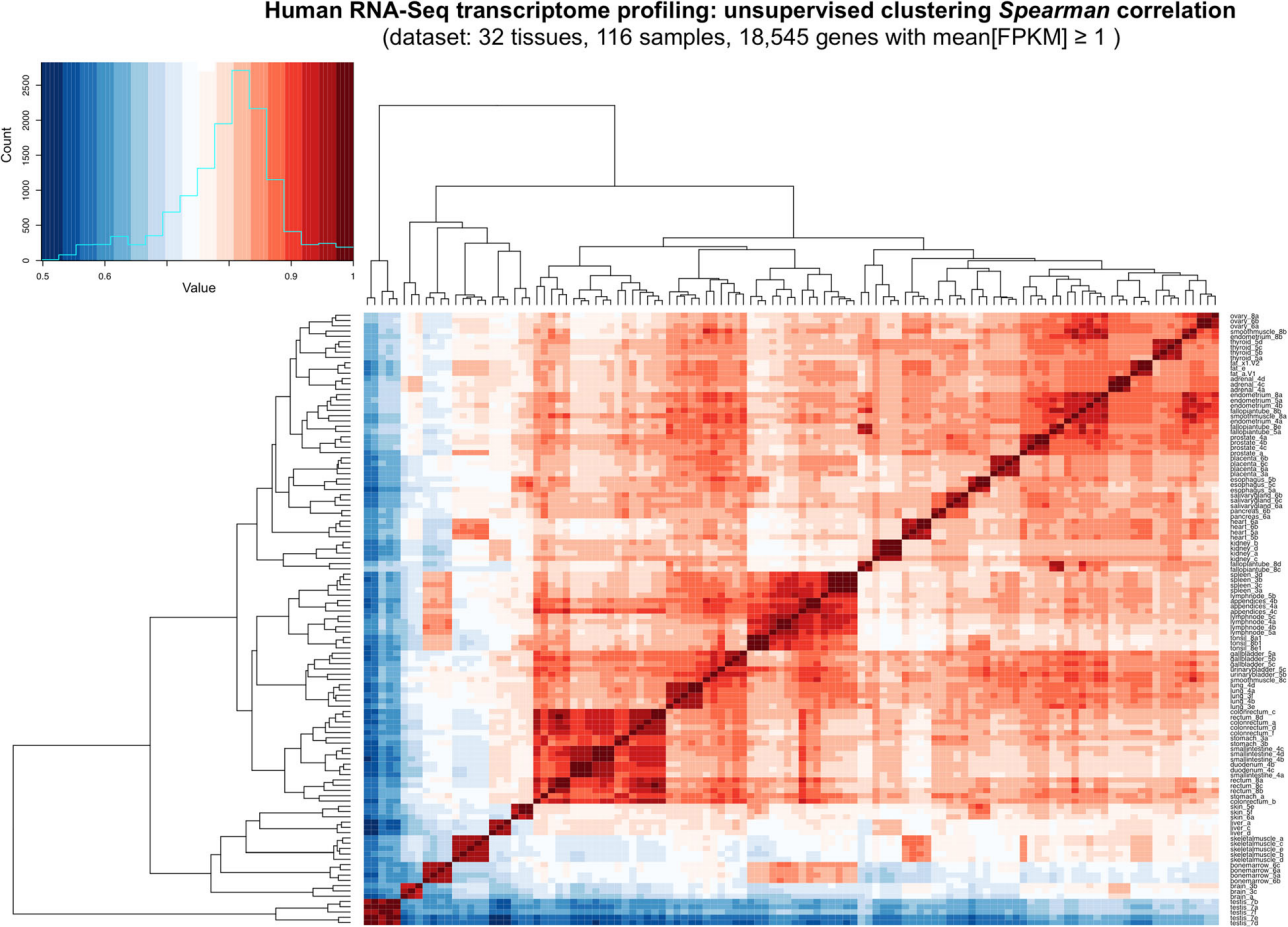
Heatmap presenting the comparison of the transcriptomic profiles of 32 human tissues. Unsupervised clustering analysis of the global expression correlation along 116 samples of 32 human normal tissues. The main plot presents the color heatmap and dendrogram produced by the comparison of all the expressed genes (18,545) along all the samples. The pair-wise distances between samples are calculated using the values of: 1 – *Spearman* correlation coefficient. The color bar with scales shows dark-red corresponding to minimum distances (i.e. maximum correlation) and dark-blue to maximum distances (i.e. minimum correlation)

RNA-Seq expression data from all the tissue samples, taken as normalized FPKM (Fragments Per Kilobase of transcript per Million mapped reads) from [3], were log2 transformed to obtain the final expression values as: log2(FPKM + 1). Normalized expression distributions of these samples can be seen in Additional file 1: Figures S1 and S2. Unsupervised clustering of the samples based in all genes expression was done using agglomerative hierarchical clustering (with the *hclust* R function) and calculating the distances based on: [1 – *Spearman_correlation*]. This clustering was done for all the 116 samples (Fig. 1) and just for the 32 tissues using the average expression of the replicates (Additional file 1: Figure S3). The coexpression dataset was built calculating the pair-wise *Spearman* correlation coefficient (r) of all the genes along the 116 samples and only selecting, as positive gene-pairs, the ones with a correlation coefficient ≥ 0.85. Crossvalidation of these correlation values was applied by a random selection of two sample replicates from each tissue (i.e. 32 × 2 = 64 samples) and recalculating again the *Spearman* correlation for these random subsets of the data. This sampling was done 100 times, annotating for each gene-pair the number of times that its r coefficient was ≥ 0.85. Only the gene-pairs validated 100 times in this sampling were selected. In this way, a final set of highly correlated gene-pairs was produced that included 2298 genes and 20,005 coexpression interactions. This coexpression dataset is provided as Additional file 2, indicating the names of all the gene-pairs and their correlation value. A gene coexpression network derived from the coexpression data was built using Cytoscape (www.cytoscape.org) and we applied the MCODE algorithm to identify clusters inside the network. This algorithm performs an analysis of the topology of the network to find densely connected regions that define modules. The coexpression network built with Cytoscape including all the subnetworks found (with information about the specific proteins in each), as well as the parameters derived from the graph analysis, is provided in Additional file 3.

### Evolutionary analyses

For the evolutionary analysis and determination of *Lowest Common Ancestor* (LCA), we used a database of orthologous proteins: Orthologous MAtrix (OMA, http://omabrowser.org/) [19, 20]. OMA includes a database and resource with methods for the inference of orthologous among complete genomes [19, 20]. We downloaded the OMA database into a local MySQL database and created Python scripts to search for the Ensembl ID's from our transcriptomic data into this local database and to calculate the LCA into each respective ortholog group. In this way we obtained a table with the number of protein-coding genes assigned to each clade in the human taxonomy lineage, as defined by the Taxonomy resource from NCBI (*Taxonomy ID* 9606 for human, *Homo sapiens*; database accessed 9 January 2016). Furthermore to produce a time-scale, we integrated these data with the TimeTree of life (www.timetree.org) [21, 22], that includes the tree of living species calibrated to time. The analysis of the number of genes placed along the evolutionary time-scale allowed visualization of the profile of human genes origin for the whole genome (genome-wide) or for specific subsets of genes. In this genes/time profile we calculated the number of protein-coding genes that had LCA corresponding to each clade (or level) in the taxonomy lineage –that for human includes 31 consecutive levels– and we identified certain levels where major changes occur. These are taken as most significant stages and proposed as key *evolutionary hallmarks* including specific sets of the human protein-coding genes that are identified.

### Functional enrichment analysis and identification of gene modules

For the functional enrichment analysis we used DAVID (david.ncifcrf.gov) [25] and GeneTerm-Linker (gtlinker.cnb.csic.es) [26] bioinformatic tools with the list of genes from each evolutionary stage level of the human lineage. In all cases the enrichment analyses were done using a hypergeometric test and adjusting the p-values for multiple testing with the Benjamini-Hochberg procedure [27]. In the same way, we also investigated the functional enrichment of the subnetworks generated by the clusters and modules found in the analysis of the gene coexpression network.

### General calculations and statistics

All the data analyses and graphics have been produced in the R statistic environment. General functions as *boxplot*, *image, qplot (*from *ggplot2 library)* or *wilcox.test*, have been applied over the different data presented. Some specific methods or algorithms are cited along different sections of this manuscript.

## Results and discussion

### Human global transcriptome profile based on expression in multiple tissues

The unsupervised clustering analysis of the global expression correlation along 116 samples of 32 human normal tissues is presented in Fig. 1. This plot shows the color heatmap and dendrogram produced by the comparison of all the expressed genes (18,545) along all samples. The pair-wise distances were calculated as: (1 – *Spearman* correlation coefficient); that is a non-parametric approach to calculate similarity and distance. In this way, the heatmap shows a clear relationship between the samples from the same tissue (i.e. the biological replicates come together) and also presents the proximity between the tissues that have strong biological and physiological links, such as: spleen, lymph nodes and tonsils (all related to the lymphatic system); or stomach, duodenum, small intestine, colon and rectum (all related to the digestive system). A color bar with scales for the heatmap is included in Fig. 1, to indicate that dark-red corresponds to minimum distance (i.e. maximum correlation) and dark-blue to maximum distance (i.e. minimum correlation). White color corresponds to medium values and the distributions inside the color bars show that the major part of the comparisons have values around *r* = 0.80–0.85 (i.e. pale-red colors). The results also reveal that some tissues have transcriptomic profiles very different to the rest, being testis the most different one that produces a clearly separated branch in the dendrogram.

### House-keeping and tissue-enriched genes

**Fig. 2:**
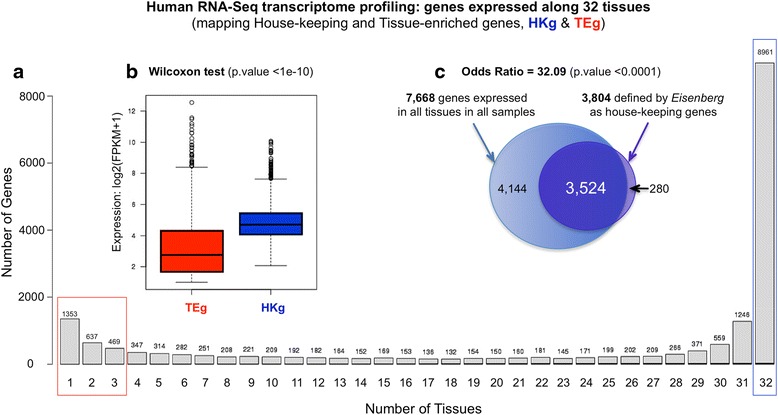
Number of genes expressed along 32 tissues derived from RNA-Seq transcriptomic data. **a** Plot showing the number of genes expressed, with FPKM equal or higher than 1, per number of tissues. The house-keeping genes are labeled as HKg and tissue-enriched genes as TEg. **b** Comparison of the expression distributions of HKg *versus* TEg. **c** Venn diagrams showing the intersection of 7668 genes (expressed in all the biological replicates of all tissues) with the dataset of 3804 house-keeping genes obtained from Eisenberg and Levanon (2013) [28]

The transcriptomic data allowed creation of two relevant subsets of genes: House-keeping (HKg) and Tissue-enriched (TEg) genes. Figure 2a shows the number of genes expressed, with FPKM equal or higher than 1, per number of tissues. This calculation allowed the identification of 8961 ubiquitous genes present in at least one replicate of all tissues, and 7668 genes expressed in all the 116 samples (i.e. in all the biological replicates of all tissues). The intersection of these 7668 genes with a curated dataset of 3804 house-keeping genes created by Eisenberg et al. [28] gave a total of 3524 HKg (Fig. 2c), indicating a large overlap of 93 %. To identify how relevant was this, we calculated the odds ratio (OR) of such overlap and obtained a very significant value of OR = 32.09 (with 95 % confidence interval, CI, of the OR = 28.27–36.43). For the tissue-enriched genes we explored the other side of the data in Fig. 2a and considered just the genes that were expressed in only one, two or three tissues (2459 genes). We did not take only one, but also two or three tissues, because some tissues are physiologically very related and in fact presented high correlation between them, for example: colon and rectum; small intestine and duodenum, etc. These observations indicated that such tissues share a large number of common genes in their expression profile. Finally, a global comparison of the expression distributions of HKg versus TEg indicated that the Tissue-enriched genes showed significantly lower expression values than the House-keeping genes (Fig. 2b). To demonstrate this difference, we made two statistical tests, t-test and Wilcoxon. In both cases, the p-values were very low (p-value < 1e-10) and the difference between the mean expressions of TEg and HKg was 1.61 (in the log2 scale). Thus, we can conclude that this difference is not by chance but due to a real different expression regulation that should be considered in further analyses. These boxplots also show that the global variability of TEg (which presents values below 1 and above 12 in the log2 scale) was larger than the global variability of HKg. This observation indicates that the TEg present a larger range of expression values along different tissues and the HKg have a tighter regulation.

### Human gene hallmarks on the evolutionary time-scale

For the evolutionary analysis, we used a phylostratigraphic method for reconstruction of macro-evolutionary trends based on the principle of “founder gene” formation [10]. Typically, these methods first identify the homologues of a given gene and then use the divergence between the two most distant to determine the gene age. Historically, such studies have been using BLAST [29] for homology searches. However, this approach was shown to introduce some biases into the analyses [30, 31]. Another approach is to use orthologous groups to determine the age of a gene. Orthologues are believed to be functionally more similar than paralogues [32] and by definition they trace back to an ancestral gene that was present in a common ancestor of the compared species [33]. The parameters used for clustering orthologous groups affect the age estimations for a gene; for example, restrictive parameters tend to limit the set of possible progenitors [9]. Nevertheless, both approaches used for dating the gene origin depend on the correct identification of homologues and/or orthologues, but in the second case the accurate reconstruction of orthologous families imposes a higher stringency and it implies a conservation along the evolutionary clades.

**Fig. 3:**
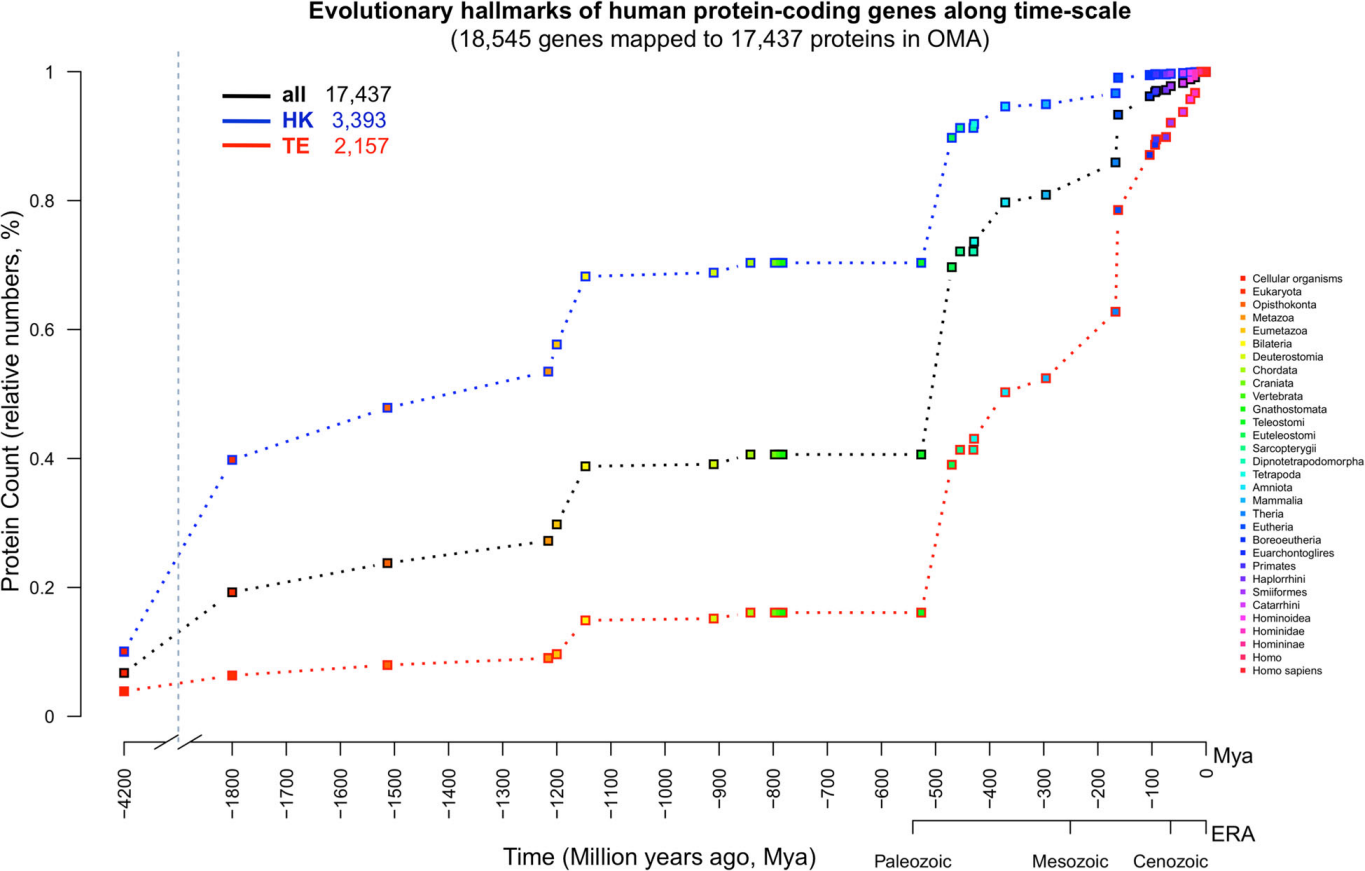
Evolutionary hallmarks of human protein-coding genes along time-scale. Plot presenting the number of human protein-coding genes (in relative terms to the total, %) that are assigned to each of the 31 taxonomic clades (labeled in colors on the right). For each one of these taxonomy levels the graph represents the cumulative percentage of protein-coding genes that are dated at such level. In this way, the 31 taxonomic clades are placed as dots along the time-scale (in million years ago, Mya) from the origin to present. Line in black includes all the 17,437 proteins derived from the mapping of expressed genes in OMA. Line in blue includes only the HK genes: 3393. Line in red includes only the TE genes: 2157

For our analysis, we identified the group of orthologues that corresponded to each of the 18,545 genes detected in the transcriptomic study, mapping them to the corresponding human protein-coding genes in the OMA database [20] (i.e. mapping of 18,545 genes to 17,437 proteins), and then assigning the *Lowest Common Ancestor* (LCA) to each human protein according to its orthologous family. The use of OMA in comparison with BLAST homology approach gives us a more detailed view of gene origin since, as we indicated above, it uses a more restrictive grouping method. Thereby, the number of genes dated on ancient clades is lower. Once we identified the LCA for each human protein-coding gene we assigned such protein/gene to the corresponding taxonomy level in the human lineage as defined in NCBI (http://www.ncbi.nlm.nih.gov/Taxonomy/Browser/wwwtax.cgi?mode=Info&id=9606), that includes 31 taxonomic groups as consecutive clades from the first one, named *cellular organisms*, to the last one *Homo sapiens*. Figure 3 presents these 31 taxonomic clades placed along the time-scale (in million years ago, Mya) from the origin to present. For each one of these taxonomy levels we represented the cumulative percentage of proteins that are dated at such level. This is done in the following way: first, plotting all the genes mapped to OMA proteins (Fig. 3, black line in the graphic, that includes 17,437 proteins); second, the same plot is produced but including only the proteins that correspond to House-keeping genes (Fig. 3, blue line includes 3393 proteins, HK); third, plot including only the Tissue-enriched genes (Fig. 3, red line includes 2157 proteins, TE).

**Fig. 4:**
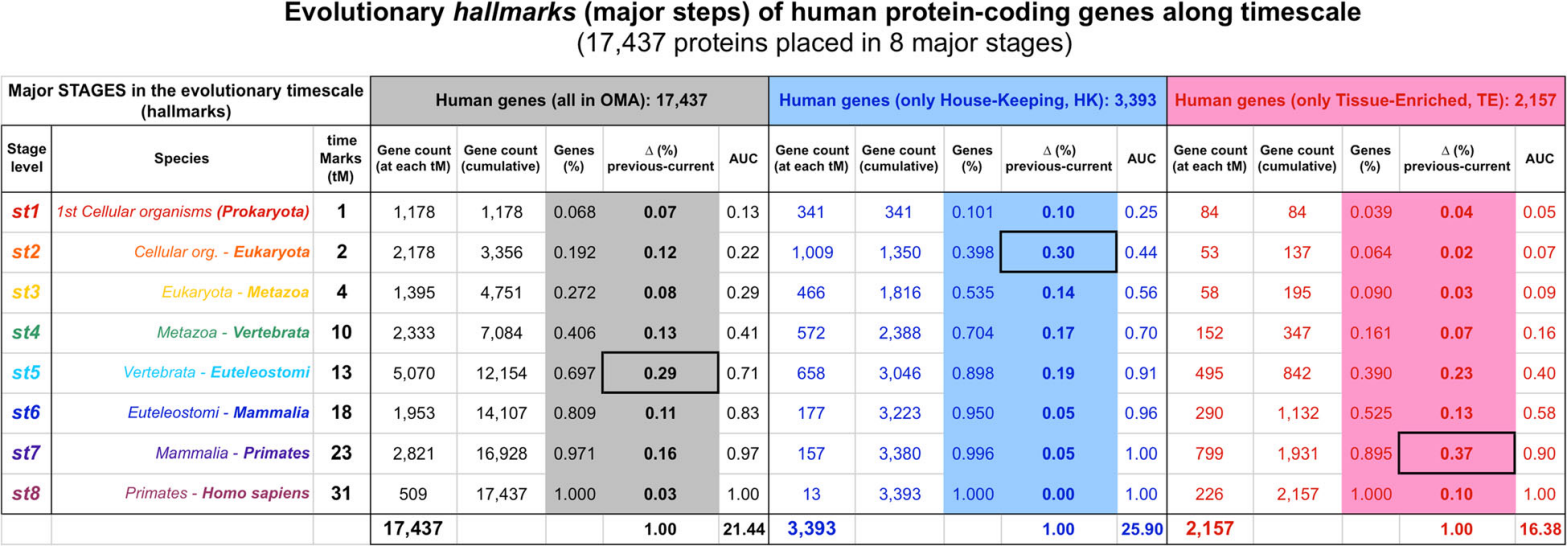
Evolutionary hallmarks of human protein-coding genes along time-scale. Table showing the numbers corresponding to the human proteins found at each of the eight major evolutionary steps depicted from placing them on the time-scale. These eight stages provide the evolutionary hallmarks of the human proteome. The stage levels correspond to: **st1)** *Cellular organisms* (*Prokaryota*); **st2)** *Cellular organisms* to *Eukaryota*; **st3)** *Eukaryota* to *Metazoa*; **st4)** *Metazoa* to *Vertebrata*; **st5)** *Vertebrata* to *Euteleostomi*; **st6)** *Euteleostomi* to *Mammalia*; **st7)** *Mammalia* to *Primates*; and **st8)** *Primates* to *Homo sapiens.* The numbers are presented for all the 17,437 protein-coding genes, for the 3393 HK genes and for the 2157 TE genes

The analyses of these plots obtained with the phylostratigraphic method revealed the presence of some major differential steps on the emergence of protein-coding genes along the evolutionary time-scale from origin to present. Looking at all the expressed coding genes, we can see the global evolutionary profile of the organism (human, in this case), but along this profile we can identify some more prominent steps in the accumulated relative number of genes (genes count) along time. For example, looking at the plot of the HK a large increase is observed at the start of the curve, from the first taxonomy level (origin, *Cellular organisms*) to second (*Eukaryota*) taxonomy level (i.e. the first phylostratum *Cellular organisms* - *Eukaryota*). By contrast, the TE have the major emergence of genes much later (around the *Mammalia*) (Fig. 3). The complete timeline includes 31 phylogenetic clades (named in the figure legend), but by analysing these points it was possible to identify eight major steps or stage levels (named “hallmarks”) that appear on the human gene profile along evolutionary time. Moreover, we could assign the number of human protein-coding genes that emerged along each one of these eight stages for the three categories reported: all the expressed protein-coding genes mapped to OMA, the HK and the TE. The eight stage levels identified are: **st1)** *Cellular organisms* (*Prokaryota*); **st2)** *Cellular organisms* to *Eukaryota*; **st3)** *Eukaryota* to *Metazoa*; **st4)** *Metazoa* to *Vertebrata*; **st5)** *Vertebrata* to *Euteleostomi*; **st6)** *Euteleostomi* to *Mammalia*; **st7)** *Mammalia* to *Primates*; and **st8)** *Primates* to *Homo sapiens.* All the numbers corresponding to the human protein-coding genes assigned to each of these eight evolutionary hallmarks are included in Fig. 4, that indicates how many are in each stage either considering the complete human gene set or just the HK or the TE. All the information about each one of the 17,437 human protein-coding genes including the assignment to stages is also provided as Additional file 4.

The analysis of the hallmarks also reveals that the HK genes are more ancient than TE genes. The plots in Fig. 3 and the data in Fig. 4 show clear difference between them. The HK genes present a major increase or expansion in stage 2 (*Prokaryota* to *Eukaryota*), with 1009 genes and a change of ≈ 30 % with respect to the total. By contrast, the TE genes show a major increase in stage 7 (*Mammalia* to *Primates*) with 799 genes and a change of ≈ 37 %. These observations seem to indicate that house-keeping genes emerged early in evolution and are older in age, knowing that they reflect more essential and constitutive functions. The idea that gene essentiality is associated with older genes has been reported in several studies, for example on yeast [14] and mammalian genes [16]. By contrast, the observations that human tissue-specific genes had emerged later in evolution may reveal that human specific cellular or physiological roles are implemented at molecular level by the appearance of newer genes.

### Gene age data comparison

As we indicated above, there are some studies that use the phylostratigraphic method to explore the age of human genes, but most of these studies use sequence similarity search (with algorithms like BLAST) to look for the oldest homologues to the human [12, 16, 34]. To compare the results on human gene age assignment done in this work with other available age assignments, we took the published data from Domazet-Lošo (2008) [12] and from Neme (2013) [34], and we represented the information about allocation to Lowest Common Ancestor (LCA) of the human genes in phylogenetic clades of the evolutionary tree. The assignments were done using 15 common phylostratum to allow the comparison of the data. The results of this comparison are included in Additional file 1: Figure S4 and they show a general similarity but some important differences. The most significant difference corresponds to the fact that both Domazet-Lošo [12] and Neme [34] placed a very large number of genes on the first stage of the evolutionary time-scale that goes from the origin of life to first *cellular organisms* (i.e. pre-*eukaryota*): 8285 of 22,845 (36 %) [12] and 7309 of 22,154 (33 %) [34]. This result denotes a bias that, as we indicated above, can be due to the methodology of using the homology search approach. In any case, the idea suggested that one third of the human proteoma may have emerged in evolutionary time before the origin of eukaryotic cells needs deeper studies and it is not what we observed in our analyses.

Another important difference is that the proposed age mapping allocates the largest number of genes, first, to the *Chordata-Vertebrata-Euteleostomi* phylostratums (with 5070 genes) and, second, to the *Mammalia-Eutheria* (with 2172 genes) (Additional file 1: Figure S4). From the evolutionary point of view these results make a lot of sense since the time-scale of life [21, 22] reveals two large expansions of the species precisely around the vertebrates time (between 600 and 400 Mya) and around the time of the mammals appearance (between 250 and 100 Mya). These expansions are well reflected in our time-scale profile (Fig. 3). Finally, it is important to indicate that the age mapping presented in our study only considers human protein-coding genes that are included in orthologous families (mapping a total of 17,437) and in this way it has a lower coverage over human genes than the other reported studies which include more than 22,000 genes in each case [12, 34].

### Functional enrichment of the genes at different evolutionary hallmarks

We performed functional enrichment analyses of the sets of protein-coding genes included in each one of eight major stages found in the evolutionary study. The full results of these analyses are provided as Additional file 5. In all the stages the functional enrichment makes clear biological sense and provides a strong support to the allocation of many biological processes in evolutionary time. In brief, we comment and discuss below some interesting functions enriched in each stage.

Stage-1, from the origin of life to first cellular organisms (i.e. *Prokaryota*). This stage comprises the genes occurring over two major domains of life: *Archaea* and *Bacteria*. Determining the LCA, our data shows that human has 6.76 % (1178) of the protein-coding genes assigned to *Prokaryotic* age. *Prokaryotes* are organisms that lack both membrane-bound organelles and nucleus. Functional enrichment analysis showed that this stage involved many basic metabolic processes like glycolysis (GO:0006007, glucose catabolic process), the Krebs cycle (GO:0006099, tricarboxylic acid cycle), and lipid oxidation (GO:0009062, fatty acid catabolic process). The enrichment also shows the appearance of the oldest cellular organelle, the mitochondria, and the oldest macromolecular machine, the ribosome, that are well reported to be dated to *Prokaryotic* times.

Stage-2, Cellular organisms to *Eukaryota*. According to basic literature, the defining feature of eukaryotic cells is that they have membrane-bound organelles, especially the nucleus, which contains the genetic material, and is enclosed by the nuclear envelope. Protists, fungi, animals, and plants all consist of eukaryotic cells. *Eukaryotic* cells also contain other membrane-bound organelles such as the Golgi apparatus. *Eukaryotic* organisms can be unicellular or multicellular. The functional enrichment analysis for the 2178 genes that emerged along this stage showed well the formation of the principal complexes expected in *Eukaryotes*. In this way it is remarkable the enrichment on nuclear pore proteins, nuclear import proteins, nucleosome and chromatin proteins, as well as many proteins involved DNA and RNA activity: mRNA and rRNA processing, mRNA splicing, DNA unwinding, DNA polymerase, DNA/RNA helicase. This stage also marks in time the appearance and biogenesis of some major molecular complexes: the proteasome (GO:0005839, proteasome core complex), the spliceosome, and the ribosome (at this stage mainly the proteins of the large subunit RPLs, in contrast to the ribosomal proteins of the small subunit RPSs, that were mostly allocated to *Prokaryotic* age).

Stage-3, *Eukaryota* to *Metazoa*. The third stage comprises organisms from *Opisthokonta* and *Metazoan* clades with 1395 protein-coding genes (27.25 % cumulative). The *Opisthokonts* are a broad group of eukaryotes, including both the animal and fungi kingdoms, sometimes referred to as the “fungi/metazoan group” [35]. This stage comprises metazoan, fungal and protistan taxa, and other multicellular taxa (such as plants, and red and brown algae) [36]. They also include known fungi and/or parasites of plants like: *Ascomycota, Basidiomycota, Chytridiomycetes, Glomeromycota, Microsporidia, Urediniomycetes, Ustilaginomycetes* and *Zygomycota* [37]. According to our functional enrichment analysis, this stage involves different genes responsible for signal transduction like the GTPases. Some of the enriched terms are: pyrophosphatase activity, nucleoside-triphosphatase activity, GTP binding, transferring phosphorus-containing groups. All these functions indicate that it may be the time when phosphorus and phospate acquired a key role in protein function and regulation. Other enriched functions, like post-translational protein modification, calcium-binding EF-hand, protein transport and localization also indicate cellular protein regulation.

Stage-4, *Metazoa* to *Vertebrata*. This stage includes organisms from *Eumetazoa, Bilateria, Deuterostomia, Chordata, Craniata* and *Vertebrata* with 2333 genes (40.63 % cumulative). The main novelties of this stage are the appearance of protein kinase activity, and the presence of growth factors and some specific signaling proteins like WNT. All biochemically characterized members of the WNT superfamily encode enzymes that transfer organic acids, typically fatty acids, onto hydroxyl groups of membrane-embedded targets [38]. Other enriched terms in this stage, like sarcomere and contractile fiber part, may indicate the emergence of the muscular structures present in vertebrates [39].

Stage-5, *Vertebrata* to *Euteleostomi*. This stage represents the largest step in the human lineage according to the number of protein-coding genes assigned (5070), that correspond to a 29 % of the total. The stage comprises organisms from *Gnathostomata* (jawed vertebrates), *Teleostomi* (bony fish and tetrapods) and *Euteleostomi* (bony vertebrate) [40]. The enrichment analysis shows a large functional expansion including new biological systems, like the neural and the vascular-circulatory systems, represented in enriched terms like: neurogenesis, neuron differentiation, axogenesis, voltage-gated channels, neuromuscular junction development, blood vessel development, vasculature development, mesenchymal cell development and differentiation, etc. Many other genes are assigned to biological regulation and regulation of cellular processes; including cell death and apoptosis. Finally, the appearance of the large family of homeobox proteins seems to be placed at this stage.

Stage-6, *Euteleostomi* to *Mammalia*. In this stage there are organisms from *Sarcopterygii* (lobe-finned fishes) [41], *Dipnotetrapodomorpha* (new taxon from NCBI comprising lungfishes), *Tetrapoda* (four-legged vertebrates), *Amniota* (comprising the reptiles, birds and mammals that lay their eggs on land or retain the fertilized egg within the mother) and up to *Mammalia* clades. With 1953 genes at this stage, the human lineage achieves 80 % of its gene composition. The most relevant enriched terms are related to the hematologic system, marking the appearance of the leukocytes and the lymphocytes. Previous phylogenetic analyses based on gene expression data also placed the date of many proteins from leukocytes around the time of the mammals’ clade [42].

Stage-7, *Mammalia* to *Primates*. This stage comprises clades of *Theria, Eutheria, Boreoeutheria, Euarchontoglires* and *Primates*, representing organisms that give birth to live young without using a shelled egg up to placental mammals [43]. There are 2821 genes emerged on this stage, adding up to 97.08 % of the cummulative profile in the human gene lineage. A large amount of these genes are enriched in the terms: regulation of gene expression and transcription. Other more specific terms are related to the skin (epidermal and epithelial cell differentiation, keratinization) or with the sexual reproductive system (male gamete generation, spermatogenesis and sexual reproduction). This stage also includes a family of cytochrome P450 proteins (that are around 23) and the mammalian defensins (that are 6): DEFA1B, DEFA3, DEFA4, DEFA5, DEFA6, and DEFB4A. Defensins are a family of antimicrobial peptides and vital contributors to host immune response. Being constitutive or inducible expressed genes, they have been shown to contribute to innate host defense via direct bactericidal activity, as well as to adaptive immunity through effector and regulatory functions [44].

Stage-8, *Primates* to *Homo sapiens*. The last stage of human development, with 509 genes, presents a group of quite specific functions played by specific protein families, such as: somatotropin hormone, cytochrome P450, GTPase activator activity, defense response to fungus and bacterium provided by histatins. HIS1 and HIS3 (histatin proteins) have been found only in saliva of humans, macaques and some other primates but not in any other mammals [45]. They are a family of histidine-rich polypeptides that probably function as part of the non-immune host defense system and appeared very late in evolution [45]. Cytochromes P450 constitute a superfamily of proteins that existed in virtually all species from prokaryotes to humans. Most of these proteins in the CYP1, CYP2, CYP3 and CYP4 families encode enzymes involved in the metabolism and elimination of potential toxic compounds like drugs or foreign xenobiotics, and are inducible by various environmental stimuli [46]. This last stage includes a small subset of six cytochromes P450 that seem to be very specific of the primates-human clades: CYP2A7, CYP2C9, CYP2D6, CYP2J2, CYP2S1 and CYP3A43. The appearance late in evolution of some of these genes may reflect their functional specificity and it is known that they play a key role in human health [46].

### Network analysis reveals evolutionary age conservation of coexpressed proteins

**Fig. 5:**
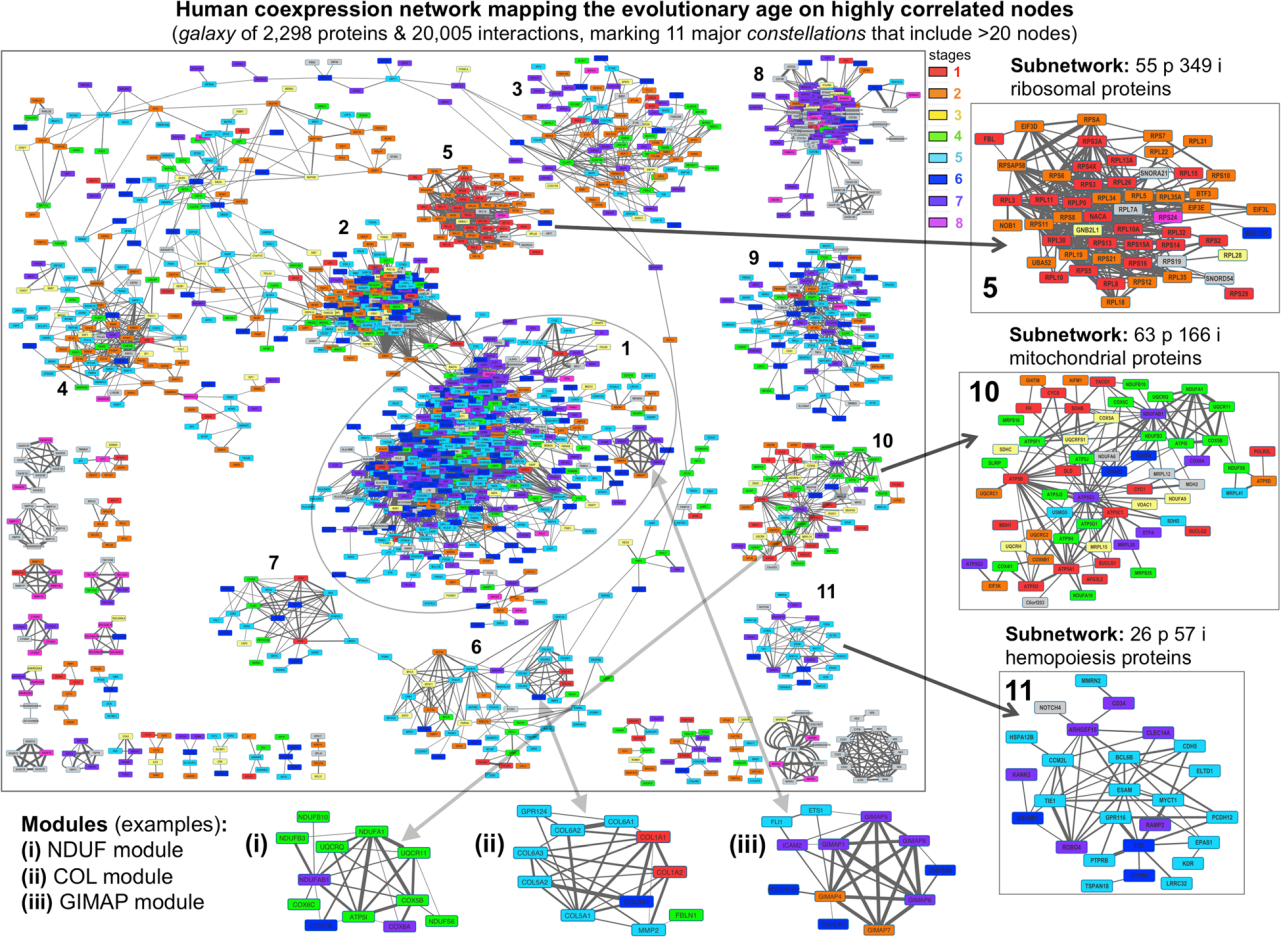
Human coexpression network mapping the evolutionary age on highly correlated nodes. Representation of the coexpression complex network –like a *galaxy*– that includes 1691 protein nodes related with 19,615 interactions. This network corresponds to a subset of the larger coexpression network produced, which included 2298 proteins and 20,005 interactions (provided in Additional file 2). The subset is done to include only the groups that had at least five linked proteins. The color mapping of the nodes correspond to the eight stages that were identified in the evolutionary study (as reflected in the labels included at the top right). The network also includes numbers for 11 major subnetworks –clusters of closely related proteins that include more than 20 nodes– considered as major *constellations* in the *galaxy* of nodes. Three panels on the right show an enlarged view of three subnetworks corresponding to: ribosomal proteins (5), mitochondrial proteins (10) and angiogenesis proteins (11)

The global coexpression analysis of the human protein-coding genes allowed the construction of a network including highly correlated protein pairs. The integration of these data with the data from the evolutionary analysis –that provided the identification of the eight stages along evolutionary time– did allow mapping the age of the genes on the network according to such stages. These results are presented in Fig. 5 that shows a complex network –like a *galaxy*– that includes 1691 protein nodes associated by 19,615 interactions. This network corresponds to a subset of the coexpression data, build as indicated in Methods, which included 2298 proteins and 20,005 interactions (this full coexpression data is provided in Additional file 2, and the network in Additional file 3). The subset is done with only the groups that had at least five linked proteins, since we wanted to provide in Fig. 5 a visible representation of the network with a clear color mapping of the eight stages that were identified in the evolutionary study. The colors of the stages are also presented in the illustrated table, Fig. 4, to allow better identification of the number and % of proteins at each age hallmark. The analysis of the network (Fig. 5) done with the algorithm MCODE revealed the existence of 11 major subnetworks, which can be considered as major *constellations* in the *galaxy* of relational nodes. The color code of this large graph indicates that there is enrichment in certain colors in each subnetwork. To prove this in a more accurate way, we built a graphic representation for each one of the 11 subnetworks found to show the proportion of proteins assigned to each of the eight evolutionary stages with their corresponding color code (colors as in Figs. 4 and 5). This graphic is presented in Additional file 1: Figure S5, that shows each subnetwork with its specific color pattern, indicating that there are always some predominant colors: subnetwork 5 is the oldest with red predominant colors and subnetwork 11 is the newest with blue predominant colors. As a conclusion, these results revealed that in the groups of highly coexpressed proteins there is a tendency to include proteins of the same evolutionary age.

Finally, we did a functional enrichment analysis of the proteins forming the 11 subnetworks which again showed a coherent biological enrichment in specific functions: (subnetwork 1) immune response; (2) cell cycle; (3) cytoskeleton; (4) RNA splicing; (5) ribosome; (6) extracellular matrix; (7) muscle and contraction; (8) gametes and reproductive process; (9) cell junction and cell adhesion; (10) mitochondria and ATP synthesis; (11) angiogenesis and vasculogenesis. More detailed results for this analysis are included in Additional file 1: Figure S6. Thus, we observed that age-related proteins are predisposed to present expression coregulation and to have close functional links.

Combining age data, functional data and coexpression data can provide a deeper view about the links and roles of the human protein-coding genes. In this way, we observed for example that subnetwork 5 (which contains proteins related with ribosome and translation) presented as expected an overwhelming majority of ancient genes from the *Prokaryotic* or *Eukaryotic* age (stages 1 and 2). On the other hand, subnetworks 1 (immune response, leukocyte/lymphocyte activations), 8 (gametes and reproductive process) and 11 (angiogenesis and vasculogenesis) showed a higher proportion of recent genes dated after *Vertebrata* (Additional file 1: Figure S5). These results agree with studies based on yeast protein physical interaction networks, arguing that proteins preferentially interact with proteins of same age and origin [47]. Moreover, it was previously shown that coexpression networks can be conserved over the evolutionary history, and these genes tend to be functionally related and provide selective advantages [48]. It has been also reported that coexpression networks are found associated to functions like cell adhesion, cell cycle, DNA replication and DNA repair [49], and this is in agreement with functions found enriched in subnetworks of our analyses: subnetwork 2 and 9 (Additional file 1: Figure S5).

## Conclusions

The transcriptomic study of the human gene expression distributions and profiles along 32 tissues provided a global mapping of the activity of most human genes and of the links between them, showing the expected association of samples from common physiological regions, i.e.: the gastrointestinal tract (stomach, duodenum, small intestine, colon and rectum), the hematopoietic and lymphatic system (bone marrow, lymph node, spleen, tonsil and appendix) and the muscle (cardiac and skeletal muscle).

The evolutionary study of the human protein-coding genes placed them in the time-scale of the living species and revealed eight distinct hallmarks along such time-scale (i.e. eight major steps), showing that the HK genes are more ancient than the TE genes. The HK genes present the major emergence in stage 2 of the evolutionary profile, while the TE genes have the major emergence in stage 7. The functional enrichment study found coherent groups of terms and annotations assigned to the genes placed at each evolutionary stage. For example, in stage 1 there were many functional terms on essential metabolic processes, like aerobic respiration and mitochondrial activity; and in stage 2 there were enriched functions related to the nucleus and genome regulation, like chromatin and nucleosome assembly, DNA replication, mRNA processing.

Finally, the study of the pair-wise correlation of the gene expression profiles along tissues allowed building human gene coexpression networks and find modules with functional and biological meaning. The mapping of the age of the protein-coding genes on these networks demonstrated the existence of tight links between age-related proteins.

## Additional files

Additional file 1: Additional PDF file that includes six supplementary figures. (PDF 3633 kb)
Additional file 2: TABLE (.XLS) including the values of the gene pair-wise *Spearman* correlation and the cross-validation from the coexpression analysis, as well as the gene IDs to allow the reconstruction of a human coexpression network with 2298 proteins and 20,005 interactions. (XLSX 1048 kb)
Additional file 3: Cytoscape file (format.cys, produced with Cytoscape version 3.1.0) that includes the complete coexpression network that is produced in this work and presented in Fig. 5 of the main article. This is a file to be opened only with Cytoscape program. (CYS 2316 kb)
Additional file 4: TABLE (.XLS) including the data and IDs of the 17,437 human protein-coding genes included in each of the eight evolutionary stages. (XLSX 1432 kb)
Additional file 5: TABLE (.XLS) including the results of the functional enrichment analysis of the 17,437 human protein-coding genes included in each of the eight evolutionary stages. The results for each of the eight stages are presented in different spreadsheets. (XLSX 99 kb)
