# Supplementary material for: Evolutionary hallmarks of the human proteome: chasing the age and coregulation of protein-coding genes
Source: BMC Genomics. 2016 Oct 25;17(Suppl 8):725. doi: 10.1186/s12864-016-3062-y (PMC5088522; doi:10.1186/s12864-016-3062-y)
Supplement: Additional file 1: — Additional PDF file that includes six supplementary figures. (PDF 3633 kb) [file 12864_2016_3062_MOESM1_ESM.pdf]

Additional File 1

Evolutionary hallmarks of the human proteome: chasing the age and coregulation of protein-coding genes

Authors:

Katia de Paiva Lopes, Francisco José Campos-Laborie, Ricardo Assunção Vialle, José Miguel Ortega, Javier De Las Rivas

Corresponding authors: Dr. Javier De Las Rivas, jrivas@usal.es

This PDF file includes 5 supplementary Figures:

Supplementary Figure 1: Density plots representing the distributions of gene expression signals in 32 tissues corresponding to 116 samples (i.e. mean distribution of the replicates from each tissue) obtained with RNA-Seq in log2 scale. The RNA-Seq data distributions present a bimodal shape that reveals the existence of two components assigned to high and low expression genes. The expression signal represented corresponds to the log2 of the FPKM+1.

Supplementary Figure 2: Box plots of the expression signal from each one of the RNA-Seq samples studied. The distributions of expression values represented correspond to the log2 of the (FPKM+1) signal for each one of the 116 samples analysed. In total 32 different human tissues are included.

Supplementary Figure 3: Clustering of human tissue expression profiles. Heatmap and clustering of the Spearman correlation for 18,545 genes from 32 human tissues (pair-wise comparison). A color bar with scales for the heatmap is included indicating that dark-red corresponds to minimum distance (i.e. maximum correlation) and dark-blue to maximum distance (i.e. minimum correlation). White color corresponds to medium values and the distribution inside the color bars shows the density of compared tissue pairs present at each correlation value range.

Supplementary Figure 4: Comparison of different studies on the evolutionary origin of human genes. The plot represents the same data included in the table and both show a comparison of the assignment of human protein-coding genes to the Lowest Common Ancestor (LCA) in phylogenetic clades of the evolutionary tree. The assignments were allocated to 15 phylostratums to allow the comparison of the data. The data in **blue** are obtained from the work of **DomazetLoso_2008** (12); the data in **red** are obtained from the work of **NemeTautz_2013** (34); and the data in **green** correspond to the present work (**Lopesetal_2016**).

Supplementary Figure 5: Relative composition on proteins from different ages in 11 subnetworks found in the human coexpression network. Graphic plot representing, for each one of the 11 subnetworks found in the coexpression network, the proportion of proteins assigned to each of the 8 evolutionary stages. The stages are marked with their corresponding color code indicated in the label.

Supplementary Figure 6: Human coexpression network: functional enrichment of major subnetworks. Table showing a summary of the results from the functional enrichment analyses done with the proteins included in each of the 11 subnetworks labeled at the right and included in the network provided in **Figure 5** in the main article. The number of proteins (p) and interactions (i) that each subnetwork includes are also indicated.

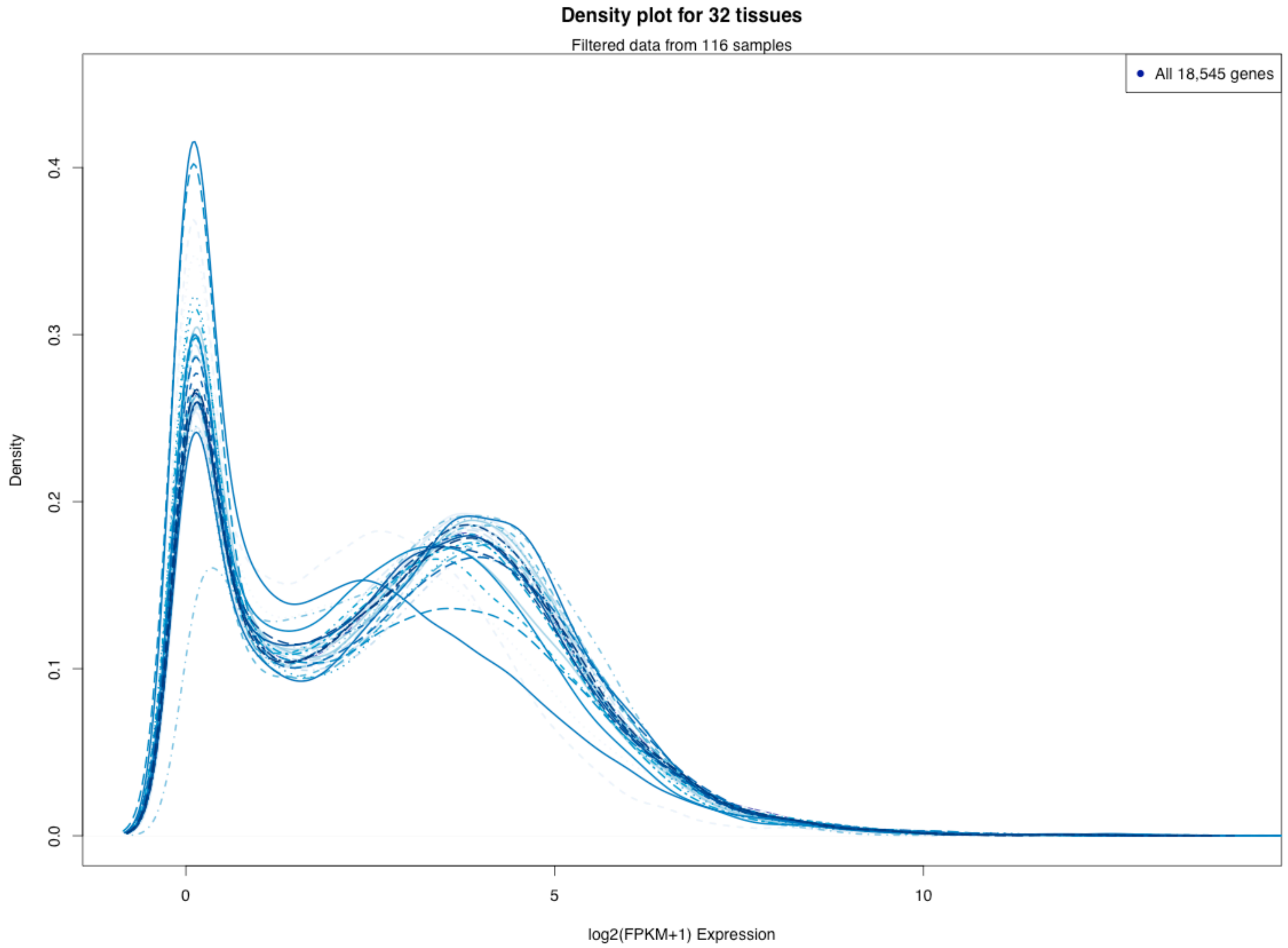

Supplementary Figure 1. Density plots representing the distributions of gene expression signals in 32 tissues corresponding to 116 samples (i.e. mean distribution of the replicates from each tissue) obtained with RNA-Seq in log2 scale. The RNA-Seq data distributions present a bimodal shape that reveals the existence of two components assigned to high and low expression genes. The expression signal represented corresponds to the log2 of the FPKM+1.

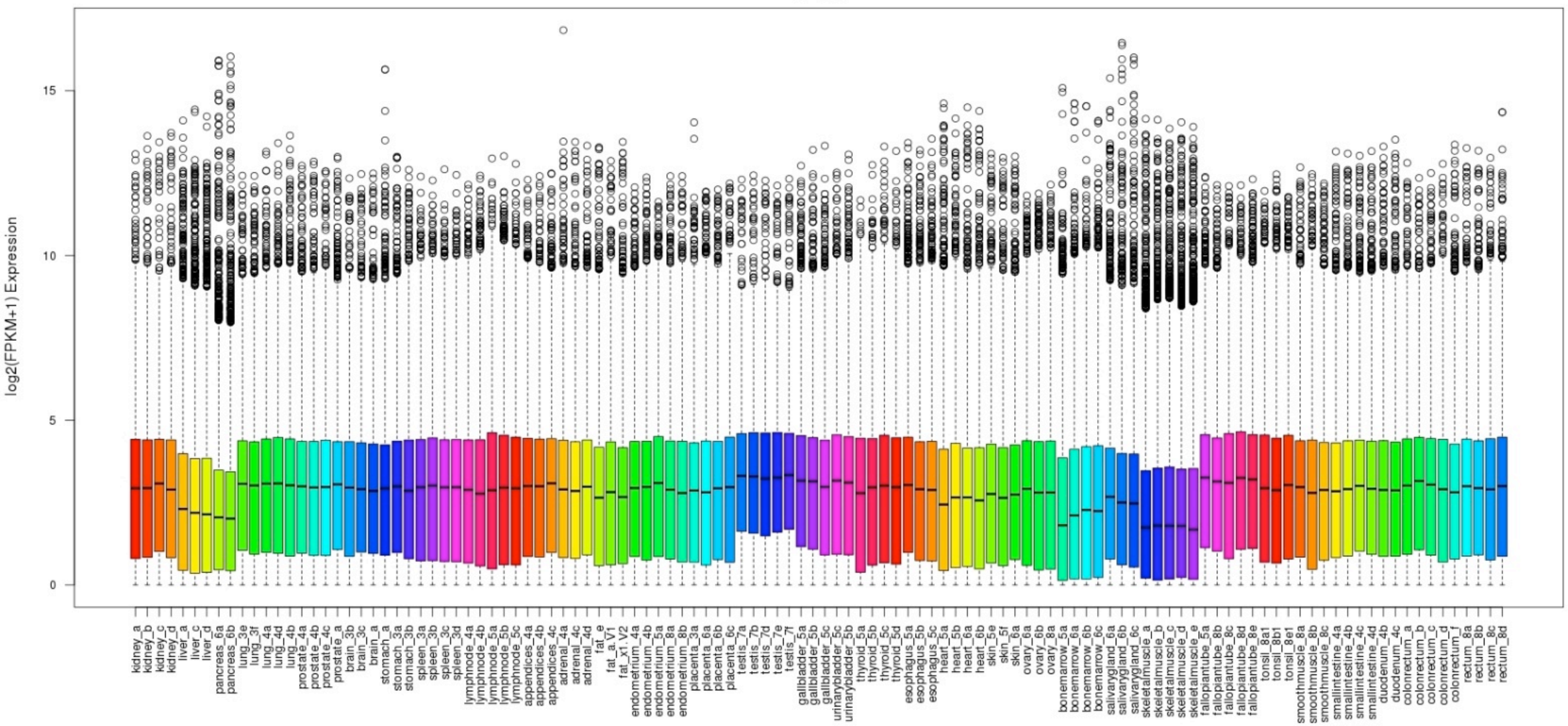

Supplementary Figure 2. Box plots of the expression signal from each one of the RNA-Seq samples studied. The distributions of expression values represented correspond to the log2 of the (FPKM+1) signal for each one of the 116 samples analysed. In total 32 different human tissues are included.

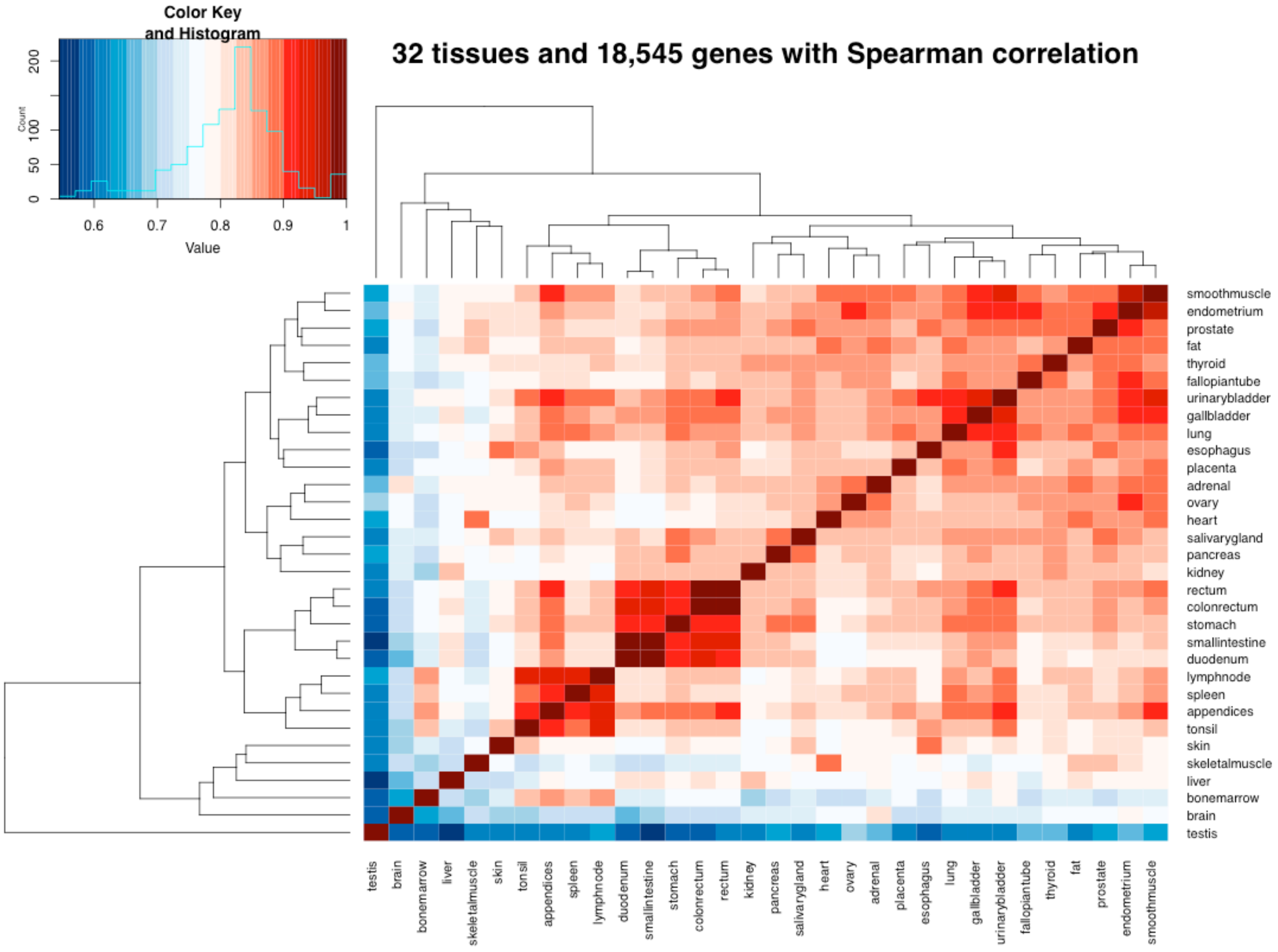

Supplementary Figure 3. Clustering of human tissue expression profiles. Heatmap and clustering of the Spearman correlation for 18,545 genes from 32 human tissues (pair-wise comparison). A color bar with scales for the heatmap is included indicating that **dark-red** corresponds to minimum distance (i.e. maximum correlation) and **dark-blue** to maximum distance (i.e. minimum correlation). White color corresponds to medium values and the distribution inside the color bars shows the density of compared tissue pairs present at each correlation value range.

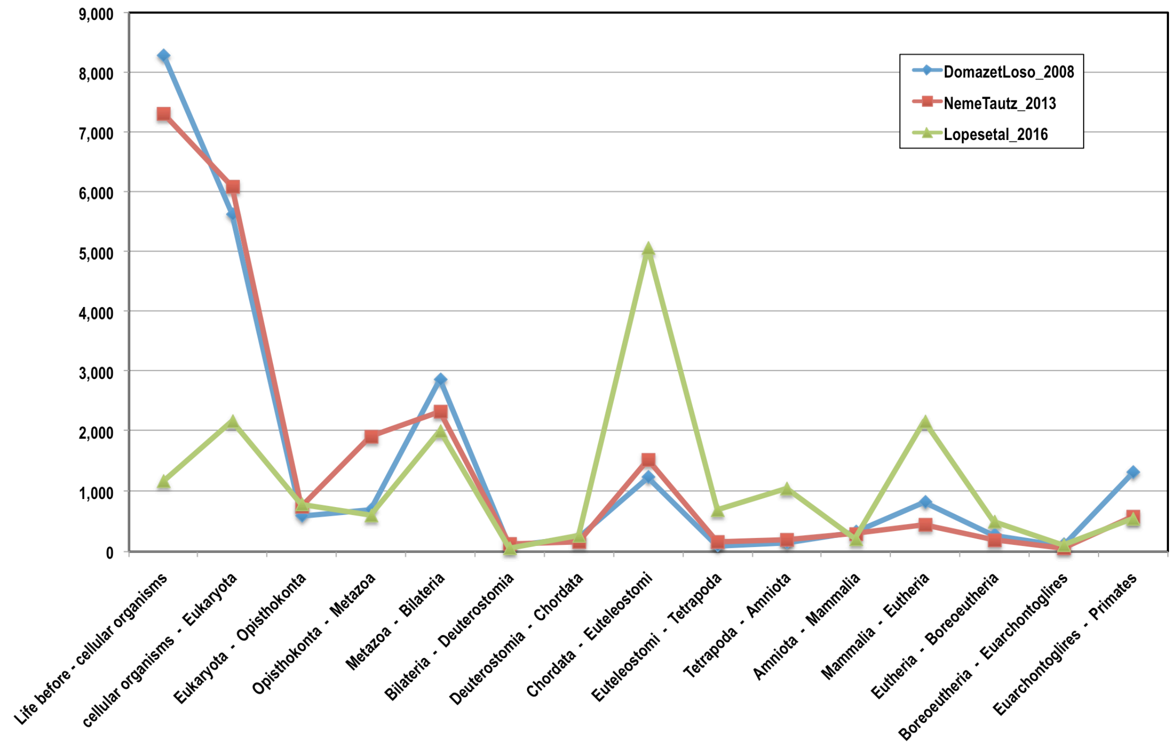

Assignment of Lowest Common Ancestor (LCA) of human genes	DomazetLoso_2008	NemeTautz_2013	Lopesetal_2016
**phylogenetic_clades (phylostratum)**	**number_g_ensembl**	**number_g_ensembl**	**number_g_ensembl**
Life before - **cellular organisms**	8,285	7,309	1,178
cellular organisms - **Eukaryota**	5,633	6,092	2,178
Eukaryota - **Opisthokonta**	600	750	791
Opisthokonta - **Metazoa**	703	1,918	604
Metazoa - **Bilateria**	2,875	2,337	2,012
Bilateria - **Deuterostomia**	93	128	57
Deuterostomia - **Chordata**	244	162	264
Chordata - **Euteleostomi**	1,247	1,531	5,070
Euteleostomi - **Tetrapoda**	106	161	693
Tetrapoda - **Amniota**	157	197	1,053
Amniota - **Mammalia**	335	292	207
Mammalia - **Eutheria**	830	448	2,172
Eutheria - **Boreoeutheria**	285	189	500
Boreoeutheria - **Euarchontoglires**	124	57	110
Euarchontoglires - **Primates**	1,328	583	548
	**22,845**	**22,154**	**17,437**

Supplementary Figure 4. Comparison of different studies on the evolutionary origin of human genes. The plot represents the same data included in the table and both show a comparison of the assignment of the human protein-coding genes to the Lowest Common Ancestor (LCA) in phylogenetic clades of the evolutionary tree. The assignments were allocated to 15 phylostratums to allow the comparison of the data. The data in **blue** are obtained from the work of **DomazetLoso_2008** (12); the data in **red** are obtained from the work of **NemeTautz_2013** (34); and the data in **green** correspond to the present work (**Lopesetal_2016**).

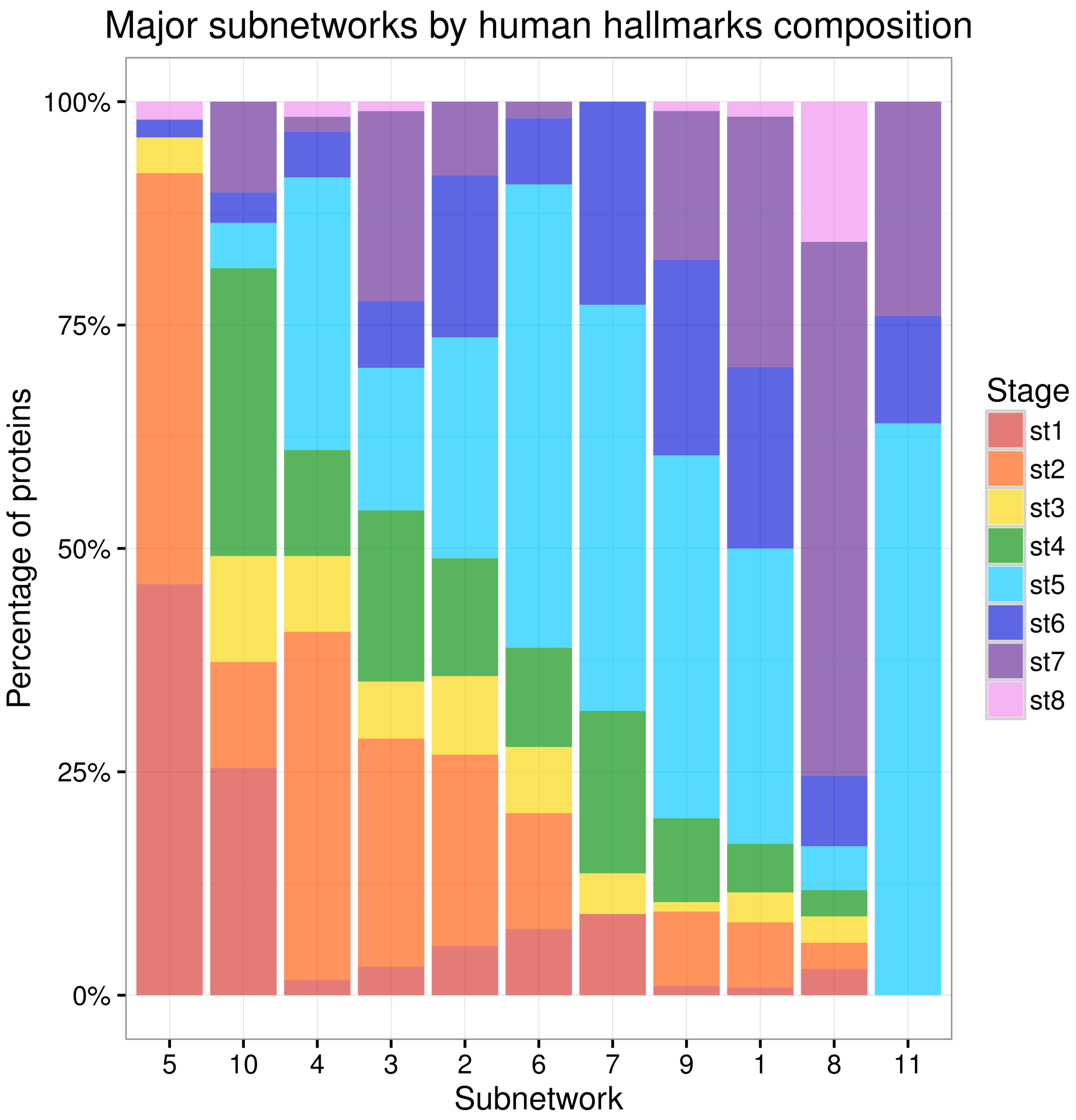

Supplementary Figure 5. Relative composition on proteins from different ages in the subnetworks found in the human coexpression network. Graphic plot representing, for each one of the 11 subnetworks found in the coexpression network, the proportion of proteins assigned to each of the 8 evolutionary stages. The stages are marked with their corresponding color code indicated.

Human coexpression network mapping the evolutionary age on highly correlated nodes

(*galaxy* of 2,298 proteins & 20,005 interactions :: functional enrichment of 11 major subnetworks that include >20 nodes)

Functional enrichment: Gene-Ontology (GO) terms	P-value	Z-score	Combined Score	subnetworks
leukocyte activation (GO:0045321)	5.39E-49	-2.37	248.26	c1: 517p 7364i
lymphocyte activation (GO:0046649)	6.01E-42	-2.32	205.61	
activation of immune response (GO:0002253)	1.26E-41	-3.45	303.74	
antigen binding (GO:0003823)	9.93E-10	-4.70	73.40	
cytokine receptor activity (GO:0004896)	2.31E-13	-2.40	54.84	
mitotic cell cycle (GO:0000278)	3.72E-76	-2.30	383.91	c2: 194p 5919i
nuclear division (GO:0000280)	2.10E-49	-2.31	244.42	
mitotic nuclear division (GO:0007067)	7.61E-45	-2.27	217.77	
regulation of cell cycle process (GO:0010564)	1.69E-32	-2.45	165.68	
cellular component assembly involved in morphogenesis (GO:0010927)	2.46E-30	-2.34	147.20	c3: 98p 210i
microtubule-based process (GO:0007017)	1.64E-15	-2.42	71.37	
microtubule-based movement (GO:0007018)	1.45E-14	-2.29	63.09	
cytoskeleton-dependent intracellular transport (GO:0030705)	3.40E-10	-2.24	39.74	
tubulin binding (GO:0015631)	2.28E-05	-2.43	14.73	
RNA splicing (GO:0008380)	3.02E-29	-2.34	139.84	c4: 69p 199i
mRNA processing (GO:0006397)	1.01E-26	-2.39	130.33	
mRNA splicing, via spliceosome (GO:0000398)	2.22E-21	-2.22	95.33	
regulation of RNA splicing (GO:0043484)	3.86E-10	-2.13	37.44	
translational initiation (GO:0006413)	1.98E-79	-2.18	383.77	c5: 55p 349i
translation (GO:0006412)	1.18E-70	-2.33	366.97	
ribosomal subunit (GO:0044391)	1.78E-77	-2.11	365.26	
ribosome (GO:0005840)	5.20E-53	-2.23	262.17	
extracellular matrix organization (GO:0030198)	1.49E-19	-2.38	89.10	c6: 53p 117i
extracellular structure organization (GO:0043062)	1.57E-19	-2.38	89.09	
collagen metabolic process (GO:0032963)	3.82E-18	-2.17	75.29	
muscle system process (GO:0003012)	2.83E-11	-2.32	42.53	c7: 22p 59i
regulation of muscle contraction (GO:0006937)	1.52E-08	-2.26	29.65	
regulation of heart contraction (GO:0008016)	4.92E-08	-2.29	28.64	
regulation of muscle system process (GO:0090257)	5.78E-08	-2.29	28.57	
spermatogenesis (GO:0007283)	2.46E-08	-2.52	32.43	c8: 149p 4343i
male gamete generation (GO:0048232)	2.53E-08	-2.52	32.45	
multicellular organismal reproductive process (GO:0048609)	4.55E-08	-2.53	32.19	
gamete generation (GO:0007276)	6.15E-08	-2.51	31.83	
cell-cell junction organization (GO:0045216)	1.79E-06	-2.26	15.55	c9: 102p 294i
tight junction assembly (GO:0070830)	2.42E-06	-2.22	15.29	
cell junction organization (GO:0034330)	5.6E-06	-2.28	15.16	
cell-cell junction assembly (GO:0007043)	3.33E-06	-2.15	14.79	
energy coupled proton transport, down electrochemical gradient (GO:0015985)	5.02E-33	-2.89	202.94	c10: 63p 166i
ATP synthesis coupled proton transport (GO:0015986)	5.02E-33	-2.89	202.64	
mitochondrial ATP synthesis coupled proton transport (GO:0042776)	4.51E-28	-2.93	175.66	
mitochondrial proton-transporting ATP synthase complex (GO:0005753)	1.27E-31	-2.95	202.82	
angiogenesis (GO:0001525)	2.40E-12	-2.31	47.98	c11: 26p 57i
regulation of angiogenesis (GO:0045765)	4.79E-08	-2.30	26.54	
regulation of vasculature development (GO:1901342)	8.32E-08	-2.32	26.39	
regulation of vasculogenesis (GO:2001212)	9.43E-07	-2.68	26.34	

Subnetwork 1: 517 p 7364 i
immune response
leukocyte/lymphocity activation

Subnetwork 2: 194 p 5919 i
cell cycle, cell division

Subnetwork 3: 98 p 210 i
cytoskeleton, microtubules

Subnetwork 4: 69 p 199 i
RNA splicing, mRNA processing

Subnetwork 5: 55 p 349 i
ribosome, translation

Subnetwork 6: 53 p 117 i
extracellular matrix, collagen

Subnetwork 7: 22 p 59 i
muscle system, contraction

Subnetwork 8: 149 p 4343 i
gametes, reproductive process

Subnetwork 9: 102 p 294 i
cell-cell junction, cell adhesion

Subnetwork 10: 63 p 166 i
mitochondria, ATP synthesis

Subnetwork 11: 26 p 57 i
angiogenesis, vasculogenesis

Supplementary Figure 6. Human coexpression network: functional enrichment of major subnetworks. Table showing a summary of the results from the functional enrichment analyses done with the proteins included in each of the 11 subnetworks labeled at the right and included in the network provided in **Figure 5** in the main article. The number of proteins (p) and interactions (i) that each subnetwork includes are also indicated.
